# Studying multiracial adolescents' racial self‐categorization(s): Conceptual, methodological, and analytic recommendations

**DOI:** 10.1111/jora.70307

**Published:** 2026-10-23

**Authors:** Victoria Vezaldenos, Andrew Grogan‐Kaylor, Deborah Rivas‐Drake

**Affiliations:** ^1^ University of Michigan Ann Arbor Michigan USA

**Keywords:** categorical data analysis, Multiracial, QuantCrit, racial self‐categorization

## Abstract

When Multiracial youth respond to demographic questions about their race in surveys (e.g., What is your race/ethnicity?) they must reconcile their lived experiences, consider others' expectations, and weigh various contextual factors when responding. Although such racial self‐categorization choices are a fundamental aspect of Multiracial realities, multiple empirical and analytic roadblocks limit the ability to study these choices quantitatively as an outcome and with a critical lens. In this theoretical and conceptual manuscript, we address such potential roadblocks by exploring various models suitable for categorical racial self‐categorization outcomes (i.e., logistic regression, multinomial logistic regression, structural equation models). We first explain the significance of racial‐self‐categorization(s) in the development as well as empirical study of Multiracial adolescents. Next, we describe our theoretical grounding in critical quantitative frameworks, which we use to frame our analysis of data collection and analytical modeling strategies researchers might employ when studying racial self‐categorization among Multiracial youth. These insights support the field of adolescent development by improving our understanding and development of Multiracial adolescents and advancing critical discourse that challenges “race‐neutral” and (mono)racist methodologies.

The racial group(s) to which adolescents belong and how adolescents make sense of those groupings have important implications for myriad outcomes, including academic achievement, income, housing, mental and physical health, and more (Hunter, 2007; Laster Pirtle, 2020; Merolla & Jackson, 2019; Neblett Jr., 2019; Quintana & McKown, 2008; Rivas‐Drake et al., 2014; Trent et al., 2019). Troublingly, research suggests that young people who identify with more than one racial group (hereafter, Multiracial) experience poorer psychosocial and developmental outcomes (e.g., engage in more risky behaviors and experience poorer mental health) when compared with their monoracial peers (i.e., those who identify with only one racial group; AhnAllen et al., 2006; Lorenzo‐Blanco et al., 2013; Nishina & Witkow, 2020; Root, 2003). These trends are particularly worrisome when considering that by the year 2050, 20% of the U.S. population is projected to be Multiracial (Lee & Bean, 2004). As a result, researchers have become increasingly motivated to shed light on how Multiracial youth experiences might relate to various life outcomes (Gabriel et al., 2023). Moreover, adolescence is a critical developmental period in which to study how youth experience and make sense of their racial group membership as youth are gaining greater independence, developing a sense of self, reconciling their identities, and often beginning to respond to demographic questions without the help of their parents (Cross & Cross, 2007; Erikson, 1968; Phinney, 1992; Quintana, 1994, 1998; Rivas‐Drake & Umaña‐Taylor, 2019; Umaña‐Taylor et al., 2014). Therefore, examining how Multiracial youth choose to self‐categorize their race during adolescence can offer insights that will allow researchers to address the aforementioned disparities in adjustment outcomes seen between monoracial and Multiracial groups.

Racial self‐categorization^1^ is often operationalized as the racial‐ethnic label(s) that individuals ascribe to themselves, typically measured via demographic questions in surveys (e.g., “What is your race/ethnicity?”).^2^ Among Multiracial youth, racial self‐categorization is a salient aspect of their lived experiences with race (Gabriel et al., 2023; Rockquemore et al., 2009). Whereas such demographic questions may be straightforward for monoracial youth, Multiracial youth must reconcile their lived experiences, consider others' expectations, and weigh various contextual factors when responding (Bradshaw, 1992; Campbell, 2007; Kich, 1992; Miville et al., 2005; Root, 2003; Vezaldenos et al., 2025); indeed, racial self‐categorization choices are considered a formative indicator of how Multiracial youth understand racial groupings and their place(s) within them. Although a growing number of studies assess such self‐categorization (see Binning et al., 2009; Booth et al., 2022; Good et al., 2010; Panter et al., 2009; Tabb, 2016; Vezaldenos et al., 2025), the affordances and constraints of different empirical and analytic approaches have not been interrogated. Further, there is an absence of discourse addressing how these approaches align with critical quantitative epistemologies. Theoretical and methodological scholarship is needed in order to bridge the gap between how Multiracial people, including Multiracial youth, experience and make sense of race and extant categorical data analysis techniques.

In this article, we aim to build bridges between the lived experiences of Multiracial youth, namely, their racial self‐categorizations, and the quantitative approaches typically used to assess them in order to support the advancement of interdisciplinary scholarship on the ever‐increasing Multiracial population. We begin by reviewing emerging scholarship which examines racial self‐categorization among Multiracial adolescents. As part of this discussion, we briefly review strategies researchers employ when collecting demographic race data and document the challenges and affordances of such approaches. Then, using critical quantitative frameworks as guiding frames, we discuss various approaches for statistically modeling Multiracial racial self‐categorization. That is, we elucidate ways to bring conceptual, methodological, and analytic techniques into better alignment and, ultimately, seek to encourage researchers to think more critically about the ways in which they collect racial self‐categorization data at the outset of their studies and about how to ultimately model it as an outcome.

## WHY IS RACIAL SELF‐CATEGORIZATION STUDIED WITH MULTIRACIAL ADOLESCENTS?

When Multiracial youth are asked to indicate their racial self‐categorization(s), the nature of their selections are not merely an issue of capturing a demographic characteristic to take into account when studying other processes but rather reflects a substantive area of inquiry unto itself. Researchers interested in the categories Multiracial youth ascribe to themselves are thus interested in racial self‐categorization as the *object* of study.^3^ One reason this line of inquiry is developmentally significant is that Multiracial youth negotiate differences between various ethnic‐racial group labels and their relationship to these categories from a very young age (Bradshaw, 1992; Kich, 1992; Rockquemore et al., 2009; Root, 1990). In contrast to their monoracial peers, Multiracial young people often find themselves being asked to, or *forced* to, articulate an affiliation with just one of the categories to which they might belong. Questions about one's racial self‐categorization are ubiquitous in the U.S. and thus a key mechanism by which children and adolescents, especially Multiracial youth, come to make sense of racial group boundaries and their place within them (Rivas‐Drake & Umaña‐Taylor, 2019). Indeed, adolescents of all racial identities often ask each other “What are you?” as part of getting to know each other (e.g., Rivas‐Drake & Umaña‐Taylor, 2019); however, such racial identity inquiries are so common for Multiracial individuals that they may feel the need to prepare “racial elevator speeches” to counteract the microaggressive nature of this question for their group (Heilman, 2022; Tran et al., 2016). Furthermore, although many if not most researchers operationalize race as a time invariant variable, this is not true for Multiracial respondents, as they may change the way they conceptualize their Multiracial identity, and hence the way in which they self‐categorize across time or context (Gabriel et al., 2023; Lam‐Hine et al., 2024; Masuoka, 2017; Root, 1990, 1999, 2003; Tracy et al., 2010). For instance, a Biracial Black–Latinx youth may self‐identify as “Latinx,” “Black,” and/or “Multiracial” depending on the way the question was formatted, who is asking, when they were asked, and in what context they are being prompted. Indeed, theoretical and empirical work about the conditions in which Multiracial youth and young adults racially self‐categorize in particular ways has burgeoned in the past two decades (Binning et al., 2009; Booth et al., 2022; Doyle & Kao, 2007; Good et al., 2010; Hitlin et al., 2006; Panter et al., 2009; Schaefer et al., 2024; Tabb, 2016; Tracy et al., 2010; Vezaldenos et al., 2025). While much of this work initially leveraged qualitative methods and/or was descriptive in nature, there has been a recent emergence of quantitative work examining racial self‐categorization choices of Multiracial populations both cross‐sectionally and longitudinally leveraging both public (e.g., federal, state) and private datasets. Thus, in positioning, Multiracial adolescent racial self‐categorization as the object of study, scholars have advanced the field in two key ways: (a) illuminating how individual or contextual factors influence self‐categorization choices, and (b) offering insight into how and why such choices may vary over time. Yet, across this burgeoning area of scholarship there is a dearth of discourse surrounding appropriate methodological techniques, including strategies that align with critical quantitative frames.

From a developmental perspective, although racial self‐categorization choices are certainly pertinent across the lifespan, such choices are particularly relevant during adolescence. Adolescence is a sensitive period for the development of Multiracial identity, as youth are developing the social cognitive capacities that help them think about racial categories and categorization in new ways relative to childhood (Nishina & Witkow, 2020) and to do so in relation to their peers (e.g., Schaefer et al., 2024). Although early studies of Multiracial identification (prior to the 2000s) had to counter misperceptions of youths' developmental abilities to identify as such (e.g., Rockquemore & Brunsma, 2002), contemporary research since then has underscored the importance of adolescence as a period of such identity development (Doyle & Kao, 2007; Harris & Sim, 2002; Rockquemore et al., 2009). One reason for this is structural—more opportunities to identify as Multiracial due to shifts in societal practices such as the Census (and by extension in school rosters and collection of data for national datasets) have made it more viable and normative for today's adolescents to opt for Multiracial self‐classifications (i.e., choosing to self‐select more than one race; Rockquemore et al., 2009). In addition, Rockquemore et al.'s (2009) groundbreaking review of the field laid the foundation for a lifespan view of Multiracial identity development, noting its multifinality depends on contextual and developmental capacities; she noted the possibilities of change from childhood to adolescence and from adolescence through adulthood. Thus, the field has both burgeoning empirical work and a well‐established theoretical basis which support the exploration of racial self‐categorization among Multiracial adolescents. Yet, there is a paucity of literature which offers researchers empirical and methodological guidance to execute such inquiries, especially when scholars are interested in applying a critical quantitative lens.

## THEORETICAL FRAMEWORKS: QuantCrit AND QUANT(MULTI)CRIT

In our view, the study of racial self‐categorization among Multiracial adolescents can be improved by adopting a critical lens that takes into account (mono)racism and power potentially borne by and manifest in researchers' choices.^4^ In recent years, quantitative scholars taking a critical stance have emerged with recommendations for how to leverage statistics in ways that are liberatory, in that they seek to reveal, critique, and challenge social inequities (Castillo & Babb, 2024; Tabron & Thomas, 2023). Quantitative Critical Race Theory (QuantCrit) scholars, in particular, do so by explicitly rooting their methodological recommendations in the tenets of Critical Race Theory to disrupt racism, specifically (Castillo & Gillborn, 2022; Garcia et al., 2018; Gillborn et al., 2018). QuantCrit integrates Critical Race Theory principles into quantitative methods in order to reveal and challenge racist structures while also contextualizing the lived experiences of participants in one's analytical sample (Castillo & Babb, 2024; Castillo & Gillborn, 2022; Garcia et al., 2018; Gillborn et al., 2018). In alignment with QuantCrit recommendations, the authorship team provides brief positionality statements in Appendix A.

We draw on a novel extension of QuantCrit–Quant(Multi)Crit (Vezaldenos, 2025)—that extends its existing antiracist methodological arguments by integrating tenets of Multiracial Critical Race Theory (MultiCrit; Harris, 2016). MultiCrit theory is a modification of Critical Race Theory that aims to authentically recognize Multiracial realities and challenge *monoracism*, more specifically (Harris, 2016). Monoracism refers to the discrete categorization of racial groups, the enforcement of those racial boundaries, and the privileging of identities that fall neatly within mutually exclusive categories (Harris, 2016), which ultimately erases and challenges Multiracial realities. In Vezaldenos' (2025) extension, MultiCrit principles are leveraged to inform statistical recommendations that explicitly work toward disrupting and dismantling monoracism in quantitative inquiry, resulting in 5 Quant(Multi)Crit tenets: (1) *the centrality of race, monoracism, and colorism*, (2) *numbers may perpetuate a monoracist racial categorization system*, (3) *critique (Multi)racial categories: for ‘race’ read ‘(mono)racism’*, (4) *center Multiracial voice and insight*, and (5) *using numbers for social justice*. See Table 1 for the definition of these tenets.

**TABLE 1:** Quant(Multi)Crit tenets and definitions.

Quant(Multi)Crit tenet^a^	Definition
(1) The centrality of racism, monoracism, and colorism	Monoracism and colorism are endemic and evolving across time and context thus scholars should be mindful as to how their study might be reproducing these monoracist conceptualizations of racial classification and should consider how colorism could be accounted for quantitatively.
(2) Numbers perpetuate a monoracist racial categorization system	Numbers have been, and continue to be, manipulated to invisibilize the Multiracial population in service of white supremacy; scholars should consider the ways their studies might manipulate Multiracial participants resulting in their multiple identities being erased or misrepresented.
(3) Critique (Multi)racial categories: for ‘race’ read ‘(mono)racism’	Scholars should consider the ways Multiracial experiences do and do not map onto monoracial experiences while also refusing to conflate all Multiracial experiences as the same. Researchers should be attuned to how monoracism specifically might be manifesting “behind the scenes” of the data.
(4) Center Multiracial voice and insight	Multiracial voices should be centered throughout the research process in order to challenge the racial status quo and critique the monoracial order.
(5) Using numbers for social justice	Scholars have the power to leverage their quantitative expertise in service of social justice rather than upholding and perpetuating the monoracist status quo

^a^ Quant(Multi)Critical tenets and definitions displayed here are from Vezaldenos (2025).

Given our current focus on Multiracial experiences of racial self‐categorization, in the following sections, we first briefly review common formats used for racial self‐categorization data collection and summarize the benefits and hindrances of these techniques. We offer considerations that align with Quant(Multi)Crit tenets, rooting our methodological recommendations in this framework in order to make visible and authentically capture Multiracial realities to the greatest extent possible. We elucidate and explicitly state how the different suggested analytic approaches do or do not align with a Quant(Multi)Crit perspective, acknowledging that rarely will a study sufficiently align with all five tenets. Ultimately, by rooting this work in Quant(Multi)Crit, we offer constructive suggestions that will help scholars engage in humanizing practices in research with and about Multiracial people and thus advance anti(mono)racism in their work whenever possible.

## HOW IS RACIAL SELF‐CATEGORIZATION TYPICALLY ASSESSED?

When scholars study racial self‐categorization choices, they must deeply consider how their data were or will be collected. The ways demographic data for ethnicity and race are collected have implications for the techniques researchers might be able to leverage when studying racial self‐categorization among Multiracial youth populations. Accordingly, in this section, we describe common approaches, both historic and contemporary, to collecting racial self‐categorization data. In later sections of this manuscript, we also explain how key features of these data formats lend themselves to various analytic techniques.

### Historical approaches

For much of the history of social science in the U.S., dominant norms around demographic race questions in the U.S. have marginalized Multiracial realities (Charmaraman et al., 2014; Lam‐Hine et al., 2024; Williams, 2006). Despite the fact that racial group boundaries have evolved and been redefined throughout U.S. history (see Brodkin, 1998; Lee & Ramakrishnan, 2020; Omi & Winant, 2008; Smedley & Smedley, 2005), one constant across much of social science has been the assumption that such categories should be mutually exclusive, and thus a survey respondent should only be allowed to pick *one* (Smedley & Smedley, 2005)—this idea is monoracist.

It was not until the year 2000 that the U.S. government shifted away from monoracist survey design and began to formally recognize Multiracial populations by allowing respondents to check more than one box when indicating their race on the U.S. Census (Charmaraman et al., 2014; Lam‐Hine et al., 2024; Williams, 2006). This may be one reason for the paucity of quantitative scholarship examining Multiracial experiences prior to 2000, as researchers were not asking the right questions to accurately identify a Multiracial sample (Charmaraman et al., 2014). Since then, there has been continued advocacy around, and subsequent evolution of, race data collection standards at the government level in order to better capture Multiracial realities (U.S. Census Bureau, 2024a, 2024b). For instance, in the 2030 Census ethnic groups (i.e., Arab, Middle Eastern, or North African [AMENA], and Latinx/Hispanic) will be presented alongside the list of racial groups for the first time in a check all that apply format^5^ (U.S. Census Bureau, 2024b). These updates are important, as they may ultimately shift norms around such questions in social science, because many researchers look toward government standards to determine best practices for their own data collection. These changes present additional opportunities for the study of racial self‐categorization choices among Multiracial adolescents, as public (e.g., federal, state) and private datasets that adopt these changes will have more accurately identified Multiracial participants. Although these emerging standards are beginning to take hold, it is important to also review other approaches for capturing race data that are commonly seen across disciplines.

### Contemporary strategies

Researchers employ myriad techniques when prompting respondents about their race. Some of these approaches are better than others in their ability to account for Multiracial participants. For instance, one strategy researchers sometimes use is to add an “Other” category to their list of racial group options. This approach allows for participants themselves to indicate that the racial groups listed in a survey do not align with how they identify. However, especially when asked in a “select one” format, this option presents several potential problems. For one, using the term “Other” can be harmful to respondents, making them feel that they are different and do not belong among the recognized racial groups.^6^ Second, if the goal is to identify Multiracial respondents specifically, the “Other” category does not provide helpful information. It invisibilizes—that is, effectively erases or makes invisible—the actual racial identities participants hold. It is unclear if those who select “Other” are in fact Multiracial, or selected the “Other” box for some other unknown reason. Consequently, researchers who take on this approach often drop those who selected “Other” from their analyses, or elect to treat this group as a monolith in their models. For these reasons, this approach contradicts all Quant(Multi)Crit tenets (see Table 1).

An alternative is to add an option explicitly labeled as “Biracial/Multiracial” in addition to, or in lieu of, the “Other” category. This format allows for slightly more specificity, as one could be more certain that Multiracial people would be captured within a category of their own. Nevertheless, this approach erases nuance and oversimplifies Multiracial experiences by lumping all respondents into one presumably monolithic group, thus contradicting Quant(Multi)Crit tenets 1 and 3 (see Table 1).^7^ A more ideal format—that is now more common following the Census adaptations in recent decades—is to allow all respondents the option to select all the ethnic and racial categories that apply to them. This may lead to more accurate counts of Multiracial respondents because rather than having to endorse a new “Biracial/Multiracial” category, they can instead choose to identify with all groups that they are a member of. As an additional step, researchers may also offer a write‐in format for race and ethnicity, which allows for participants to self‐categorize in their own words with as much or a little detail as they wish to share. These practices are in better alignment with Quant(Multi)Crit principles as they better acknowledge Multiracial realities by attempting to capture the heterogeneity within the Multiracial population through more accurate and comprehensive self‐categorization options (see tenets 1, 3, and 4, Table 1).

Notwithstanding the ways these approaches allow researchers to capture the nuanced identities that Multiracial respondents hold, there are challenges that arise in data cleaning and analysis. In “select all” formats, it is not uncommon for dozens of unique racial combinations to arise among one's responses. This multiplicity of response choices poses analytic challenges when the categorical variable for race may have dozens or even hundreds of mutually exclusive categories, many of which might represent less than a handful of participants (e.g., having only one participant who identifies as Black/Native American/Asian). Furthermore, write‐in format questions require extensive work following data collection, as researchers need to read, interpret, and recode responses into manageable categories for analysis.

Our discussion thus far suggests there is not one best or perfect way to collect self‐categorization data—all approaches involve trade‐offs. Some formats allow for greater visibility of Multiracial realities at the outset but pose analytic challenges (e.g., due to extremely small subgroup sizes). Further, just as each of the aforementioned formats influence how data can be analyzed, these formats may also be received differently by Multiracial respondents. Multiracial people may make different selections when presented with different survey formats (Atkin & Minniear, 2023; Campbell, 2007; Johnston et al., 2014; Tracy et al., 2010). In fact, qualitative literature has documented the salience of responding to demographic forms among Multiracial youth (Johnston & Nadal, 2010; Waller et al., 2025). It is apparent from this research that responding to questions about one's race are formative experiences for Multiracial youth that prompt deeper reflection about racial categories, their definitions, and if and how they fit within each group. Thus, although many researchers might assume that racial category choices are immutable and time invariant, studies with Multiracial people indicate they may and do alter how they represent themselves on these forms, given the context and options available.

One objective of this article is to offer researchers strategies that would allow them to better explore these phenomena quantitatively and through a Quant(Multi)Crit lens. That is, we offer conceptual recommendations that will situate the field to better understand the racial self‐categorization choice of Multiracial adolescents by leveraging several different data structures. As previously discussed, we intentionally root our recommendations for studying race as an outcome within critical quantitative approaches, as they aim to leverage statistics in ways that explicitly challenge systems of oppression (Castillo & Gillborn, 2022).

## CONSIDERING ANALYTIC OPTIONS THROUGH HYPOTHETICAL SCENARIOS

From a Quant(Multi)Crit perspective, it is important to first take stock of the data one has (or plans to have) on hand (see tenet 2, Table 1). What demographic information does one have on racial self‐categorization? How was it collected, and how might the approach to data collection honor or erase Multiracial realities? Once the answers to these questions are clarified, one can consider possible analytic approaches given the complexities and/or limitations of one's data. As a starting point, we provide two scenarios—these are hypothetical depictions that researchers might use to ascertain the strengths and weaknesses of their own data or from nationally representative and publicly available datasets.^8^ These hypothetical cases illustrate the delicate balance that critical quantitative researchers often attempt to strike between honoring the multiplicity of adolescent identities and later concerns for and prioritization of statistical parsimony that may be necessary for model specification. They further demonstrate the inherent challenges with designing a quantitative survey that honors all Quant(Multi)Crit tenets (see Table 1).

### Scenario 1: Select all that apply and then choose one

The first scenario in which it is possible to model Multiracial self‐categorizations is one that reflects an increasingly common set of circumstances: aiming to collect racial self‐categorization data as comprehensively as possible, a research team presents a list of ethnic and racial categories to youth and instructs them to select as many as they identify with (i.e., “select all that apply”). To be inclusive, the research team might even allow for a write‐in option, so that youth can describe their race in their own words if the boxes presented are not sufficient. The research team might hope that by allowing youth to select as many categories as they want and by offering a write‐in option they would have nuanced racial self‐categorization data. Yet, they also likely realize that this could result in dozens (or more) of unique racial identity combinations; later, these combinations could pose analytic challenges if there are many subgroups with very low counts. The researchers might need to face the decision to drop such cases, or alternatively, to collapse them into a broader category for analysis. As a precaution and to help with later analytic decisions, the research team might decide to include an *additional* survey question that asks youth to choose for themselves where they should be counted. Thus, youth who select more than one option initially (i.e., Multiracial respondents) are then instructed to select just one race, often framed as “the one they feel closest to” or “identify with the most.” Importantly, participants can also refuse to pick only one category (sometimes indicating this in an open‐ended “Other” box) or skip this follow‐up “choose one” question altogether (i.e., choose to retain all their multiple racial self‐categorizations).

Given that Multiracial people have frequently reported that being asked (and sometimes forced) to choose one racial identity is a microaggression (Johnston & Nadal, 2010), this dataset presents a complicated, potentially paradoxical case. “Select all” style questions align with Quant(Multi)Crit frameworks in that they challenge monoracist norms and allow for the existence of Multiracial nuance (see Quant(Multi)Crit tenets 4 and 5; Table 1). However, “choose one” questions perpetuate monoracism (see Quant(Multi)Crit tenets 1 and 2; Table 1). In addition, the research team attempts to *center Multiracial voice and insight* (see Quant(Multi)Crit tenet 4; Table 1) when they choose to provide Multiracial respondents themselves agency in how to be represented in later analyses (i.e., they can choose one or skip the question leaving their initial selections intact). Because this dataset has both “select all that apply” and “choose one” data for their respondents, it poses multiple opportunities for studying race as an outcome.

### Scenario 2: Select one, including options for other and multiracial

Another scenario entails a research team who expressly wishes to avoid making subjective decisions regarding how to collapse Multiracial adolescents' responses during analyses at a later stage of the project. They might seek to ensure that the race options they chose will capture large enough samples to be able to estimate sufficiently powered models. For these reasons, they do not allow respondents to select all race options that apply to them but rather ask participants to select a single racial category. The twist here is that they include options for “Other” and “Multiracial.” In this case, if a participant feels that the singular racial categories presented (e.g., Black, White, Asian, etc.) do not fully capture their experience, they could select “Other” *or* “Multiracial.” This would result in mutually exclusive categories that would make race data cleaning and analysis more straightforward.

However, this approach privileges statistical ease rather than the lived experience of adolescents, perpetuating monoracist categorization systems by forcing all Multiracial people to be lumped together into one category. We know that there is vast heterogeneity within Multiracial samples, especially as it pertains to the specific Multiracial combinations one holds (Charmaraman et al., 2014; Lam‐Hine et al., 2024; Vezaldenos et al., 2025). This approach forces researchers to treat the category “Multiracial” as a monolith, therefore erasing the nuances of their racialized experiences and demonstrating both the *centrality of monoracism* and one way that *numbers may perpetuate a monoracial categorization system* (see Quant(Multi)Crit tenets 1 and 2; Table 1). Despite the fact that this data structure does not align with Quant(Multi)Crit recommendations, we consider it here because it may be common in larger datasets, especially longitudinal ones that have been collected over decades. Therefore, although we do not recommend the practice in this scenario, datasets structured like the one described nevertheless present important and needed opportunities for exploration of the “Multiracial” and “Other” labels as outcomes, particularly with longitudinal data.

## POSSIBLE ANALYTIC APPROACHES FOR GIVEN SCENARIOS

Given the conditions in which racial self‐categorizations are obtained from Multiracial youth, there are three primary analytic approaches that pose opportunities for studying them as outcomes: logistic regression, multinomial logistic regression, and structural equation models for categorical outcomes. We first provide a brief overview of each technique, note the formal models and technical details that each entails, and then offer specific examples as to how each might be leveraged to examine racial self‐categorization given the primary scenarios in question (i.e., “select all that apply and then choose one” and “select one, including options for Other and Multiracial”).^9^ In our discussion, we offer sample research questions and describe how the model of choice would be leveraged to answer such questions. We give specific attention to how researchers would identify an appropriate analytic sample and clarify which racial categories would be positioned as the outcome and under what conditions. Here, we also draw on Quant(Multi)Crit principles as we aim to offer categorical data analysis approaches that are best suited to address the merits of one's research question while also recognizing the multiplicity of participants' identities. We synthesize the following methodological recommendations in both a diagram (see Figure 1) and a table (See Table 2). Figure 1 offers a decision‐tree like road map detailing how the proposed data scenarios relate to our suggested models and example research questions. Table 2 synthesizes how each methodological approach makes use of categorical outcomes, technical details (i.e., identifying analytic samples), and advantages and disadvantages of each approach.

**FIGURE 1:**
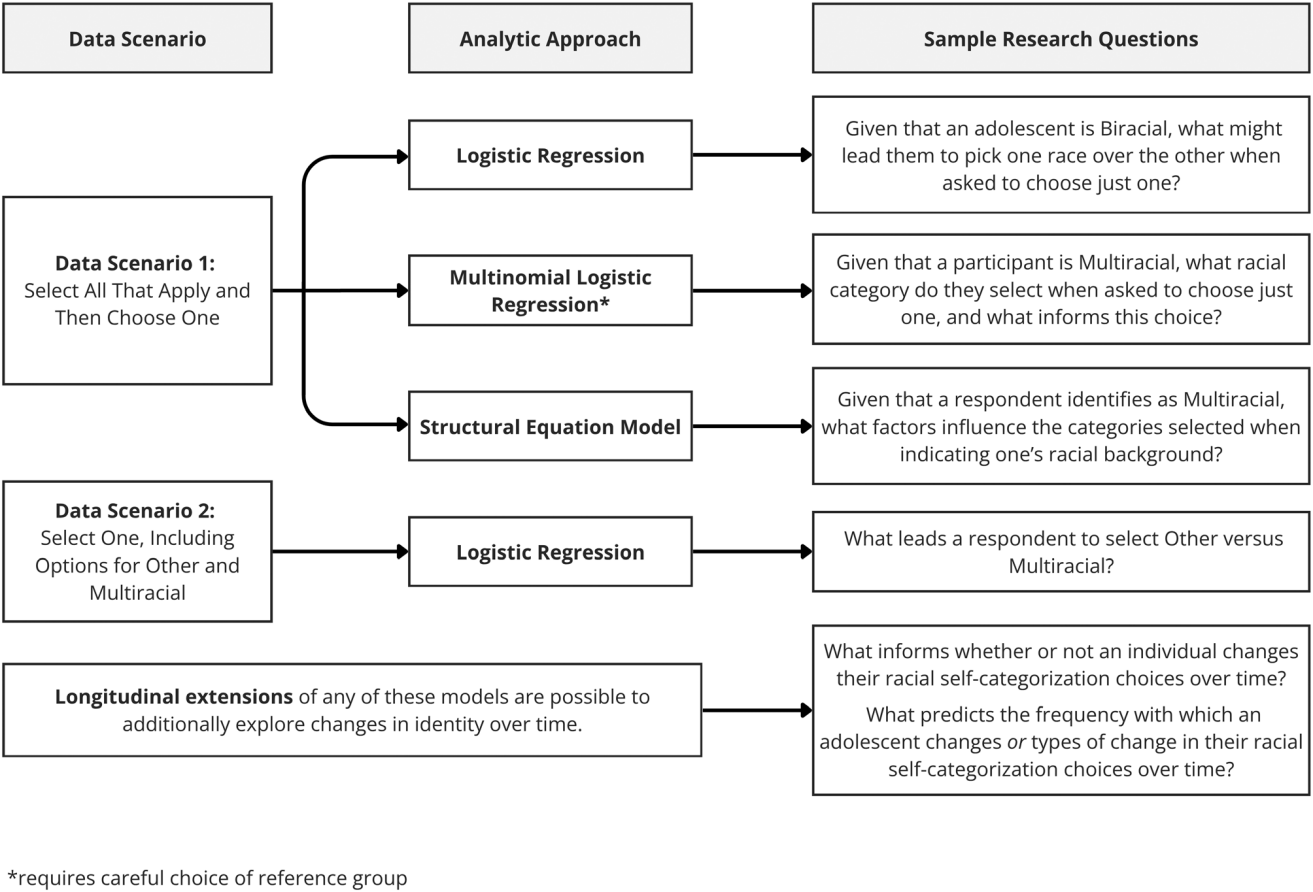
Roadmap detailing possible analytic approaches.

**TABLE 2:** Synthesis of analytic approaches: technical details, advantages, and disadvantages.

	Logistic regression	Multinomial logistic regression	Categorical SEM
How does this approach handle categorical outcomes?	Mutually exclusive dichotomous outcome (e.g., Black vs. White; White vs. non‐White; Other vs. Multiracial; Change vs. No Change)	More than 2 mutually exclusive outcomes (e.g., White, Black, *or* Asian)	More than two outcomes modeled simultaneously (e.g, White, Black, and Asian)
What does the analytic sample look like?	(Option 1) Examine small mutually exclusive subgroups (e.g., Black–White Biracial, Latinx–Black Biracial) (Option 2) Aggregate to large mutually exclusive pools (e.g. all Biracial Black respondents pooled) (Option 3) Those who selected either “Other” or “Multiracial”	Analytic samples can vary so long as participant pools have intuitive outcome categories for comparison. (e.g., all Multiracial White respondents are pooled and the outcome compares which single race they selected when forced)	Sample is all people who are Multiracial
Advantages	(Option 1) Ability to examine specific Biracial combinations (Option 2) Maximizes statistical power which can be necessary when samples of Multiracial respondents are low. Can parse out differences between pooled Biracial subgroups.	Can accommodate Multiracial (3+ races) respondents which allows for more nuanced comparisons	Does not need mutually exclusive categories as outcome Can accommodate both Biracial and Multiracial (3+ races) people simultaneously
Disadvantages	With Data Scenario 1^a^, this approach is conceptually limited to Biracial respondents. (Option 1) Need large sample sizes to explore subgroups. This might result in erasure of subgroups with too small of samples. (Option 2) May result in “double counting” participants across models (see page 20)	Need enough observations within each outcome to produce estimates which may inadvertently center whiteness and erase minoritized groups May result in “double counting” participants across models (see page 20)	Need “select all that apply” style data Because several equations are being estimated simultaneously, specifying the model and interpreting the results can become increasingly complex

^a^ Data Scenario 1: where participants were asked to select all that apply and then choose one.

### Logistic regression models: Brief overview

Logistic regression models are best suited for exploring how predictors relate to a dichotomous dependent variable (e.g., zero or one, this or that; See Table 2). When researchers are confronted with a dichotomous dependent variable (e.g., a racial category with just two mutually exclusive possibilities), logistic regression is an appropriate statistical technique that may be employed (Long & Freese, 2014).^10^

(1) $$\ln\left(\frac{p\left(\text{identit}y_{1}\right)}{1-p\left(\text{identit}y_{1}\right)}\right)=\beta_{0}+\beta_{1}x_{1}+\beta_{2}x_{2}+\ldots$$

Strictly speaking, the β regression coefficients provided by the logistic regression model estimate the association of each variable with the logarithm of the odds of the outcome. However, such regression coefficients may be intuitively difficult to understand. Thus, the coefficients from a logistic regression model can be exponentiated to create odds ratios—a comparison of odds of the outcome for two or more groups—a relatively intuitive metric of the association of the independent variable with the dependent variable. Significant odds ratios in models where racial self‐categorization is the outcome indicate that the given predictor variable is significantly associated with increases or decreases in the odds that a participant selects one of the categories over the other.

Further exploration of the logistic regression model can be carried out to estimate predicted probabilities of the outcome for various combinations of independent variables (i.e., various subgroups of individuals; StataCorp, 2025). When exploring racial self‐categorization as an outcome, predicted probabilities would indicate to the researcher the probability that a respondent—in this case, a Multiracial adolescent—would select one race over the other at different levels of the independent variable of interest.

### Applying logistic regression to scenario 1: Select all that apply, then choose one

Given that logistic regression explores a dichotomous outcome, it may be particularly useful for studying the racial self‐categorization choices of Biracial people. The researchers might ask: *given that an adolescent is Biracial, what might lead them to pick one race over the other when asked to choose just one?* Using the data resulting from scenario 1, researchers would first delineate the Biracial sample by identifying which respondents selected exactly two boxes when asked to “select all that apply.” It would then be necessary to disaggregate the Biracial sample into subgroups to explore their “select one” responses in a meaningful way. For instance, researchers should not put all Biracial youth into one model and arbitrarily code for Race 1 or Race 2 as the outcome. This would be conflating all Biracial experiences as being the same. For conceptual and sociohistorical reasons, it is inappropriate to conflate a Biracial Black–White person's selection of Black or White with a Biracial Asian‐Native American person's selection of Asian or Native American, for instance. Critical quantitative scholars can avoid these arguably illogical comparisons by grouping their Biracial sample into meaningful, mutually exclusive subgroups so that their analyses strike a balance between honoring participants' nuanced lived experiences and statistical parsimony (such approaches align with Quant(Multi)Crit tenet 3; see Table 1). We describe some of these trade‐offs and their relation to Quant(Multi)Crit theory below.

Upon disaggregating their Biracial sample into *mutually exclusive* subgroups, researchers can run a logistic regression predicting racial category selections for each subgroup. This would mean running a model for each Biracial combination. For example, in one model with all Biracial Black–White adolescents, researchers can predict what informed participants' selection of Black versus White when asked to choose just one (See Logistic Regression Analytic Option 1 in Table 2). Another model with all Biracial Latinx–Black youth can predict the selection of either Latinx or Black, and so on, for all Biracial combinations. However, this approach necessitates sufficiently large samples of mutually exclusive Biracial subgroups in order for the models to converge. Having a large enough sample of Multiracial people is a frequently cited factor that limits scholars' ability to study this population quantitatively (Charmaraman et al., 2014), so it may be unrealistic to examine mutually exclusive Biracial subgroups if one does not have enough statistical power.

This might mean that some Biracial subgroups have enough observations to produce estimable models whereas other subgroups do not. In these cases, scholars should consider what Biracial experiences they are privileging or uplifting in their analyses, and which ones they might be erasing. For instance, Biracial White people make up the largest subset of Multiracial population in the United States (U.S. Census Bureau, 2023). A dataset resulting from scenario 1 reflecting this trend in the larger population might have enough power to estimate models for participants who are Biracial Black–White, Asian–White, Latinx–White, and so on, but no other combinations (e.g., Biracial Black–Asian). This would mean the analyses would be foregrounding Biracial White experiences and ignoring data from all other Biracial people in the sample. Further, this could result in erasure of Biracial Native American and multiply minoritized Biracial respondents from the literature; populations which are already underrepresented within scholarship on Multiracial experiences (Charmaraman et al., 2014). Thus, such approaches may inadvertently contradict the intentions of Quant(Multi)Crit foundations (see Quant(Multi)Crit tenets 2 and 3; Table 1). To combat these potential issues of low sample size and erasure, scholars might elect to aggregate up to make larger pools of Biracial people for analytic purposes.

One way this could be done is to aggregate participants who share one component of their Biracial identity (see Logistic Regression Analytic Option 2 in Table 2). For instance, we have the option to create a group of all Biracial Black participants. This subgroup would include Biracial Black–White, Black–Asian, Black–Latinx, Black–Native American participants, and so on. What these participants have in common is that of the two boxes they selected, one is Black. The logistic regression model can then be used to estimate what informs participants' choice of selecting Black versus some other racial group more generally. Researchers could aggregate up for each Biracial subgroup, running a model with all Biracial Latinx, Biracial White, Biracial Asian, and Biracial Native American subgroups, respectively—indeed such an approach would be in alignment with Quant(Multi)Crit theory (see tenets 3 and 4, Table 1). Authors (2025) used this technique to examine the influences of skin color, same‐race friends, parental messages about race, and experiences of discrimination on racial category choices of Biracial high school students.

An important caveat that critical quantitative scholars must contend with is that this analytic approach results in Biracial participants being included in more than one model; something we refer to as “double counting.” For example, a Biracial White–Latinx respondent would be included in both the model of all Biracial White participants and the model with all Biracial Latinx participants. This raises concerns regarding statistical independence and may result in correlated error terms across models.^11^ However, it is important to note that any single model that takes this aggregate approach to studying Biracial subgroups *does not* itself violate the assumption of independence. However, if researchers conduct a series of logistic regressions in which Biracial respondents are included in more than one model, there may be a potential violation of the independence assumption when interpreting findings *across* models. Because respondents would be included in various combinations across the aggregate logistic regression models, researchers cannot be sure that the error terms of one model are truly independent of the others.

This might introduce bias, especially toward the means for explanatory variables as they relate to the largest subsamples of Biracial youth. Because Biracial participants would be included in two models at most, the odds ratios across models may not be independent and instead the findings would complement one another. Across models, dependent variables would be related to one another, as Biracial participants have mutually exclusive forced‐choice outcomes. In a case where a Biracial Latinx–White adolescent selected Latinx as their racial category and did not select White, the outcome variable between the Biracial Latinx and Biracial White models would indeed be correlated. However, models with correlated outcome variables are not invalid. These models are more commonly explored in econometrics work (see seemingly unrelated regressions; Cameron & Trivedi, 2009; Greene, 2018; McDowell, 2004; Zellner, 1962, 1963; Zellner & Huang, 1962). While this approach may violate the assumption of independence when looking across models, it affords researchers the ability to parse out differences in experiences between specific Biracial groups.

An analytic approach that necessitates including participants in multiple models illustrates one of the many challenges scholars adopting QuantCrit or Quant(Multi)Crit frames are presented with when attempting to conduct racially conscious work on diverse populations. Researchers must determine which statistical conventions are helpful and/or harmful when they are trying to fully represent the nuances within their sample and capture their racialized experiences quantitatively. In the case of modeling Biracial youths' racial self‐categorization, we argue that proceeding outside of the traditional statistical boundaries is worth the unconventionality in order to better convey the experiences of the Biracial population. The statistical issue (i.e., nonindependence across models) arises as a necessity to more accurately and precisely examine race as an outcome among Biracial people using logistic regression; the trade‐off is that it allows researchers to honor the multiplicity of participants' Biracial or Multiracial identities to the extent feasible with quantitative methods. While this approach does not follow statistical conventions, it privileges representation over parsimony and strikes a balance between honoring nuance and creating subsamples that have enough power to be successfully modeled and thus *uses numbers for social justice* (see Quant(Multi)Crit tenet 5, Table 1).

### Applying logistic regression to scenario 2: Select one, including options for “other” and “multiracial”

The ability to choose only “Other” or only “Multiracial” also poses an interesting opportunity for the study of racial self‐categorization among Multiracial youth in that it allows scholars to explore why a Multiracial person might choose to endorse the “Other” rather than the “Multiracial” category. The research team might ask: *What leads a Multiracial adolescent to select Other* versus *Multiracial when presented with these options?* (See Figure 1) This question would be theoretically interesting to explore because Multiracial and Other categories are often conflated with one another (Atkin et al., 2022; Gullickson & Morning, 2011; Xu et al., 2021). That is, Multiracial people may choose the Other label in “choose one” scenarios, and researchers often collapse Multiracial and Other together for their analyses. Thus, it may be fruitful to examine what factors predict the selection of either of these categories.

To examine this in a logistic regression, researchers would first create an analytic sample that contains only those who selected either “Other” or “Multiracial” (See Logistic Regression Analytic Option 3 in Table 2). Individuals who selected monoracial labels would be excluded. The “Other” and “Multiracial” labels would be the mutually exclusive, dichotomous outcome. Researchers would then explore their predictor variables of interest in relation to this outcome. This inquiry has the potential to leverage imperfect data in pursuit of social justice, such that findings might reveal significant differences between self‐selected “Other” and “Multiracial” subgroups which are often conflated (see Quant(Multi)Crit tenet 5, Table 1). This empirical knowledge would offer insight into the complexity of these respective choices and demonstrate the need to improve demographic data collection practices so that models might be more robust and better represent the lived experiences of respondents. See Table 2 for a summary of these analytic options for logistic regression along with their affordances and limitations.

### Longitudinal applications for both scenarios

There are additional applications for logistic regression for the study of racial self‐categorization over time. When data contain repeated measures of a dependent variable, multilevel logistic regression models offer one parsimonious and straightforward approach (Raudenbush & Bryk, 2010; Singer & Willett, 2003). If both of the aforementioned hypothetical scenarios involved racial self‐categorization data collected with the same participants across multiple waves, these data could be used to potentially explore: *What informs whether or not a Multiracial adolescent changes their racial self‐categorization choices over time?* (See Figure 1) Researchers would identify their analytic sample by selecting participants who identified as Multiracial on at least one occasion. For “select all that apply” style data (scenario 1) this would include participants who selected two or more boxes and for “choose one” data (scenario 2) this would include participants that selected the “Multiracial” category at least once over the course of the study.

The (in)stability of racial self‐categorization would be the dichotomous outcome variable of interest. The model would therefore explore differences among Multiracial youth who made stable selections over time and those who changed their selections at least once (i.e., no change vs. change). This inquiry can apply to both “select all that apply” and “choose one” race data in scenarios 1 and 2. For “select all that apply data,” (scenario 1) a change would be conceptualized as any alteration to the combination of boxes an adolescent checked over time. Therefore, a change could look like adolescents selecting only “Asian” at time 1 but then checking “Asian” and “Black” at time 2 (or vice versa). This could also look like a youth selecting “White” and “Latinx” at time 1 and then “White” and “Native American” at time 2. For “choose one” style data in scenarios 1 and 2, a change would be indicated by inconsistently choosing one group over time. This might look like selecting “Multiracial” at time 1 but then selecting “White” at time 2. Researchers can then estimate a model for all Multiracial respondents to assess how their predictors of interest relate to whether or not an adolescent changes their racial self‐categorization across time. This approach *centers Multiracial voice and insight* (see Quant(Multi)Crit tenet 4; Table 1) as scholars intentionally explore how Mulitracial respondents might be representing themselves over time within a particular data set. By investigating how participants alter their self‐categorizations across time researchers have the ability to critique monoracist myths of racial permanence while offering the field greater insight into the motivations that may underlie such changes.

### Multinomial logistic regression: Brief overview

At times, researchers may be confronted with a categorical dependent variable that has more than two mutually exclusive outcome categories (e.g. 1, 2, 3, etc…).^12^ When there are multiple outcome categories, and those categories are not considered to be ordered (as with racial categories), multinomial logistic regression may be appropriate (See Multinomial Logistic Regression, Table 2; Long & Freese, 2014). Briefly, multinomial logistic regression chooses one value of the outcome to be a reference category, in a process analogous to choosing a reference category for an independent variable that is to be treated as an indicator variable. Heuristically, using the example of a dependent variable with values 1, 2, and 3, one might choose 1 as the reference category. Once the reference category is chosen—usually with the lowest value by default—the procedure is then straightforward: Multiple regression equations are simultaneously estimated, comparing the probabilities of being in each of the other outcome categories, rather than the reference category (StataCorp, 2025).^13^

(2) $$\begin{array}{c} \ln\left(\frac{p\left(\text{identit}y_{2}\right)}{1-p\left(\text{identit}y_{1}\right)}\right)=\beta_{0}+\beta_{1}x_{1}+\beta_{2}x_{2}+\ldots \\ \ln\left(\frac{p\left(\text{identit}y_{3}\right)}{1-p\left(\text{identit}y_{1}\right)}\right)=\beta_{0}+\beta_{1}x_{1}+\beta_{2}x_{2}+\ldots \end{array}$$

As with the simple logistic regression model (see Equation 1 presented on page 17), regression coefficients in a multinomial logistic regression (Equation 2) are less than intuitive, here representing log risk ratios, or the logarithm of the ratio of probabilities of two different outcomes (StataCorp, 2025). Similarly to the simple logistic regression model, coefficients from multinomial logistic regression models can be exponentiated to create more intuitive ratios of the risks of two different outcomes. Also, as with the simple logistic regression model, other commands can be used to predict the probability of a specific outcome for specific groups of people (StataCorp, 2025).

When racial category is positioned as the outcome of interest, critical quantitative practice recommends that researchers deeply consider which racial group is positioned as the reference group (see Quant(Multi)Crit tenet 2, Table 1; Castillo & Gillborn, 2022; Castillo & Strunk, 2024). Although multinomial logistic regression may be conceptually straightforward, we note that the multiple sets of regression results included in a multinomial logistic analysis can be challenging to present in a parsimonious and intuitive fashion.

### Applying multinomial logistic regression to scenario 1: Select all that apply, then choose one

With an outcome that can accommodate more than two options researchers might ask: *Given that an adolescent is Multiracial, what racial category do they select when asked to choose just one, and what informs this choice?* (See Figure 1) However, critical quantitative researchers must take great care when specifying the technical components of such a model to ensure that participant lived experiences are presented in meaningful ways (see Quant(Multi)Crit tenets 2 and 3; Table 1). For instance, this approach seemingly precludes the need to run separate models for each subgroup. Thus, researchers may be tempted to have a model that pools all Multiracial participants together in which the categorical outcome is all of the potential, mutually exclusive forced‐choice responses (i.e., Native American, Black, Latinx, White, Asian, and Multiracial). Yet, issues arise when interpreting the results of such a model. In a model where the possible categories are White, Latinx, Black, Asian, Native American, and Multiracial, and where White is modeled as the reference group, for instance, we interpret the odds that a Multiracial adolescent selects Latinx/Black/Asian/Native American/Multiracial compared with selecting White. However, this comparison would not make sense for Multiracial youth in the model for whom White is not one of the categories with which they identify. Put simply, it would not make sense to model the odds of selecting Latinx compared with selecting White for Multiracial Latinx–Black–Asian participants. Therefore, similar to the logistic regression case described for scenario 1, multiple multinomial logistic regression models would need to be estimated for each Multiracial subgroup, resulting in “double counting.”

Estimating mutually exclusive subgroups would mean aggregating all Multiracial White, Multiracial Latinx, Multiracial Asian participants and so on and running separate models for each. This approach aligns with Quant(Multi)Crit principles as it has the potential to highlight heterogeneity within the Multiracial population (See tenet 3, Table 1). In practice, researchers would first identify all Multiracial White participants, which would include all respondents that selected more than one box when prompted to “select all that apply,” with the condition that one of the boxes must be White. They would then conduct a multinomial logistic regression model that includes all the Multiracial White participants to explore what factors influenced their selection of the White, Black, Asian, etc. categories when asked to “choose one.” It is important to only include the options that were actually selected by Multiracial White participants in the categorical dependent variable. For instance, if no Multiracial White respondents picked Native American when asked to “choose one,” then it should not be included as an outcome option, as the model will not be able to produce estimates.

Once the model is correctly specified with the appropriate dependent variable categories, researchers can set White as the reference group, as that was a racial category option that was relevant to all respondents in that particular (Multiracial White) model. Results would then contextualize how the independent variables in the model related to selecting each racial category in comparison to selecting White. This approach is appealing because, in contrast to the case of logistic regression, these models are able to accommodate Multiracial youth more broadly rather than being limited to those who are Biracial (aligning with Quant(Mult)Crit tenets 2, 3, and 4, Table 1). Additionally, this approach offers more nuanced comparisons between the “choose one” response options since the dependent variable can accommodate more than two categories.

Limitations of this approach include the need for enough cases in each category of the dependent variable. If nearly all Multiracial White participants selected White when asked to “choose one,” the model may struggle to produce estimates for the other category options. Therefore, this approach may inadvertently center the experiences of Multiracial White groups, and erase other Multiracial experiences, as Multiracial White people often make up the numerical majority of Multiracial samples within quantitative datasets. This unintended consequence would violate several Quant(Multi)Crit tenets (see tenets 2 and 3, Table 1). In these cases, the logistic regression approach might be preferred as it pools all other options into the second dependent variable category, thus strengthening the power for accurate estimations and offering more opportunities for the study of Multiracial subgroups with smaller sample sizes. Even with sufficiently large subsamples, this approach necessitates including participants across multiple models, which leads to the previously discussed issue of violating assumptions of independence (i.e., “double counting”). While not a fatal flaw, for the reasons mentioned above, it is a consideration that researchers should be prepared to contest if they move forward with this approach.^14^

This example application of multinomial logistic regression exemplifies the inherent tensions that come with employing a Quant(Multi)Crit lens. While the more complex approach may at first seem to offer the researcher more flexibility in representing complex identities, the implementation of the approach may be hindered by data constraints such as sample size. These real analytic roadblocks can hinder well‐intentioned researchers' ability to represent the realities of *all* the participants in their dataset, thus pointing to the need to think deeply about the trade‐offs of one's dataset, research question, and analytic approach in order to work toward social justice (see Quant(Multi)Crit tenet 5, Table 1). See Table 2 for a summary of analytic recommendations related to multinomial logistic regression.

### Longitudinal applications for scenario 1

Multinomial logistic regression models offer additional strengths for examining changes to racial self‐categorization over time with both “select all that apply” and “choose one” data (scenarios 1 and 2). The model would function similarly to the logistic regression longitudinal models but offer additional categorical options in the dependent variable. These additional categories afford researchers the opportunity to examine more nuance when it comes to studying changes to racial self‐categorization over time. With logistic regression, scholars were limited to studying two outcomes: change or no change. With a multinomial logistic regression model, researchers can examine both the *frequency* and the *types of* change over time. Thus, appropriate research questions would be: *What predicts the frequency with which an adolescent changes their racial self‐categorization choices over time*, and *what predicts the types of change in their racial self‐categorization choices over time?* (See Longitudinal Extensions in Figure 1).

As with the longitudinal logistic regression model, we recommend reducing the analytic sample to include only Multiracial respondents (i.e., those who self‐categorized as Multiracial on at least one occasion). When the dataset has three or more waves of data collection with the same participants, researchers can examine *how frequently* a Multiracial adolescent changed their racial self‐categorization across waves. They could group respondents into categories such as no change, changed once, changed two or more times, and so on. These descriptive buckets would serve as the outcome in the model and would allow researchers to examine how the predictors of interest relate to the frequency of change across time.

Additionally, multinomial logistic regression can be used to study *types of change* in racial self‐categorization across three or more waves of data collection. To do this, researchers would organize participants into subgroups based on their response patterns to the racial demographic questions. Appropriate categories might be: no change, changed away from original response and remained with the new option, changed away and then reverted back, no discernable pattern to change (e.g., changed multiple times), and so on. With these categories specified as the dependent variable, researchers can examine how different factors (i.e, individual, contextual, time (in)variant variables) relate to these patterns of race self‐categorization (in)stability among Multiracial people.^15^

It is important to note that in order for these models to estimate, one would need a sufficient number of cases in each dependent variable category. For example, if a researcher is interested in studying the frequency of change over time but only a small number of participants changed more than once, then the model would not produce reliable estimates for that outcome category. In these cases of low sample size, a logistic regression approach (i.e., one that dichotomizes the outcome to be change or no change) would be more appropriate. As with the previous longitudinal example, this approach has the potential to offer greater insight into the nuanced experiences of Multiracial respondents across time (see Quant(Multi)Crit tenet 3, Table 1). However, the feasibility of this approach may be constrained by limitations of one's dataset (e.g., sample size). This further highlights the need to strike a balance between critical quantitative tenets and what is practically feasible in exploring one's particular research question of interest.

### Structural equation modeling (SEM) for categorical outcomes: Brief overview

Whereas the previous models only allowed for mutually exclusive outcome categories, an alternative approach might be one which allows respondents to select multiple potential identities and model all options simultaneously (see Categorical SEM, Table 2). Structural equation modeling (SEM) allows the analyst to include several equations simultaneously, each with its own dependent variable (Muthen & Muthen, 2017; StataCorp, 2025; Weston & Gore, 2006). Extensions of the SEM approach to categorical dependent variables have recently become available (Muthen & Muthen, 2017). A key advantage of the SEM approach is that because there are multiple equations within a model, outcomes do not need to be mutually exclusive, and several outcomes could be selected simultaneously.^16^

(3) $$\begin{array}{c} \ln\left(\frac{p\left(\text{identit}y_{1}\right)}{1-p\left(\text{identit}y_{1}\right)}\right)=\beta_{0}+\beta_{1}x_{1}+\beta_{2}x_{2}+\ldots \\ \ln\left(\frac{p\left(\text{identit}y_{2}\right)}{1-p\left(\text{identit}y_{2}\right)}\right)=\beta_{0}+\beta_{1}x_{1}+\beta_{2}x_{2}+\ldots \\ \ln\left(\frac{p\left(\text{identit}y_{3}\right)}{1-p\left(\text{identit}y_{3}\right)}\right)=\beta_{0}+\beta_{1}x_{1}+\beta_{2}x_{2}+\ldots \end{array}$$

Structural equation models (StataCorp, 2025) for categorical outcomes have some similarities to other models for categorical outcomes in that coefficients in these models refer to the association with the logarithm of the ratio of risks of two different outcomes (see Equation 3). Here, however, the outcomes themselves are slightly different, as there is an equation for each racial identity, and each equation models the probability of choosing or not choosing that particular identity. Again, coefficients can be exponentiated to provide more intuitive measures, and other commands exist to explore the particular probability of a particular outcome for different groups of individuals (StataCorp, 2025). SEM approaches also allow one to model the correlation of the dependent variables, an important consideration here, as choices of different racial categories are likely to be correlated. This allows for greater flexibility in examining race as an outcome, especially among Multiracial populations represented with a “select all that apply” approach.

### Applying structural equation models to scenario 1: Select all that apply, then choose one

Because SEMs allow for multiple outcome variables to be modeled simultaneously, it is not suitable for “choose one” style questions. Therefore, the racial self‐categorization data in scenario 2, which only allowed participants to select one box, should not be examined with a categorical SEM. However, scenario 1 contains “select all that apply” data that *can* be leveraged to explore racial self‐categorization patterns with a categorical structural equation model. An appropriate research question for this type of data would be: *what factors influence the categories selected when indicating one's racial background?* Note that this question is much broader than the previous example research questions. This is because a categorical SEM is the only model reviewed that can allow for examining multiple box checking responses at once. To explore such a question, researchers would first specify that their analytic sample is all respondents that identified as Multiracial. Importantly, this approach aligns with Quant(Multi)Crit recommendations as it is able to accommodate *all* Multiracial people into one model, allowing for greater visibility of multiply minoritized Multiracials and those who selected three or more boxes—groups that are currently underrepresented in social science research, and within Multiracial‐centered scholarship, in particular (e.g., Quant(Multi)Crit tenet 4, Table 1). After identifying the analytic sample, researchers would then specify SEM paths between their predictor variables of interest and each of the categorical “select all that apply” options (as dependent variables) that were offered in their survey. Note that equations for these various outcomes should be correlated in the model. It is important to note that because several equations are being estimated simultaneously, specifying the model and interpreting the results can become increasingly complex so researchers should take great care when employing this technique.

Once estimated, the results will direct researchers to significant paths between the predictors and one or more of the race categories. In this case, investigators will be able to compare and contrast differences in predictor paths, meaning that they can determine which factors significantly relate to which racial category selections. This approach aligns with quantitative critical race theories as it actively challenges monoracism by leveraging an analytic approach that allows for the selection of multiple races as an outcome simultaneously. Such findings would greatly contribute to the field as it would challenge the notion that Multiracial experiences are monolithic as it can highlight for which Multiracial subgroups are predictors significantly related (see Quant(Multi)Crit tenets 1, 3, and 5, Table 1). For example, a categorical SEM where race categories are the outcome might reveal that an individual level variable (e.g., skin color) might relate to only some racial categories (e.g., Black and Latinx) but not the others, potentially revealing disparate impacts of colorism, and other predictors, on the lives of Multiracial youth (see Quant(Multi)Crit tenet 1; Table 1). See Table 2 for a summary of analytic recommendations related to categorical SEM.

## DISCUSSION

As a means to explore the experiences Multiracial adolescents draw on when making their racial self‐categorization selections, scholars can leverage logistic regression, multinomial logistic regression, and categorical structural equation models to explore how contextual and individual factors might relate to the box(es) respondents check when indicating their ethnic or racial group memberships(s). As noted, these sorts of decisions have been and will continue to be of substantive interest in the field of adolescent development (Binning et al., 2009; Booth et al., 2022; Doyle & Kao, 2007; Good et al., 2010; Hitlin et al., 2006; Panter et al., 2009; Schaefer et al., 2024; Tabb, 2016; Tracy et al., 2010; Vezaldenos et al., 2025). As the field continues to study the rapidly growing Multiracial youth population with quantitative methods, guidance aligned with critical quantitative recommendations will allow for the exploration of nuances in the Multiracial experience (Castillo & Gillborn, 2022; Vezaldenos, 2025). For Multiracial youth, the process of racial self‐categorization is often fluid and contextually dependent (Bradshaw, 1992; Campbell, 2007; Kich, 1992; Miville et al., 2005; Root, 2003; Vezaldenos et al., 2025), leading to possible lines of inquiry that are of developmental interest. Thus, the role of developmentally salient ecological processes—such as peer relations, schooling experiences, and familial socialization—can be modeled in ways that offer insight into how youth arrive at particular racial self‐categorizations. Further, the design and analytic approaches we discussed suggest multiple ways to model multiple facets of stability and change among Multiracial youth over time, and potentially across the life course; we noted ways to examine whether types of self‐categorizations change, which youth evince change at all, and what types of change are evident (e.g., stable vs. variable multiple self‐categorizations, stable vs. variable single self‐categorization, and so on). As theory and research on Multiracial youth development continues to grow, studies of their racial self‐categorization have not necessarily kept pace; the approaches we recommend in this article will potentially help us move toward better testing both foundational (Rockquemore et al., 2009) and novel (Gabriel et al., 2023) theoretical postulates, particularly as they pertain to developmental shifts in identity during adolescence.

As underscored with the data scenarios we presented, the more complex or comprehensive one's demographic data are, the more opportunities we have to model Multiracial adolescents' racial self‐categorization(s). For instance, in Scenario 1, which involves both “select all that apply” and “choose one” race data, we can leverage all three of our suggested approaches to examine racial self‐categorization both cross‐sectionally and longitudinally. Yet, the methodological approach one selects should hinge upon the research question of interest. When grounded in QuantCrit and Quant(Multi)Crit frames, researchers should consider which model, regardless of complexity, best represents the most Multiracial voices possible in one's inquiry while still honoring the research question. As illustrated in our examples, more complex models may sometimes result in the erasure of Multiracial subgroups and/or may inadvertently center numerical majorities. Complex methods do not always tell a more nuanced story, especially when trying to capture the experiences of underrepresented groups like Multiracial youth.

In full transparency, we began conceptualizing this paper to reflect the practicalities of data collection based on our own experiences grappling with challenges of designing new studies and approaching others' datasets to implement secondary analyses that address substantive questions of interest for Multiracial youth. Yet, another way to engage with the material we have presented is to start with the sample research questions posed in Figure 1 and work backward from them. That is, given one's research question, what are some feasible analytic approaches, and what would the racial self‐categorization data structure need to be to implement them? This list of sample research questions about Multiracal racial self‐categorizations is certainly not exhaustive, but we do believe they are of common interest in the literature to date and reflect what is possible to answer with extant datasets. Thus, we hope this article will be a useful reference for novice and experienced researchers alike.

The methodological and analytic suggestions outlined in this paper serve as a starting point for inquiries into the self‐categorization choices of Multiracial people. We acknowledge that there may be additional data structures, modeling techniques, and research questions that may also be suitable for exploring race as an outcome that were not addressed in the current manuscript. We also note that our suggested approaches may quickly become complicated, requiring sufficient statistical training and time to work through such challenges creatively. We further detail these limitations and offer suggestions for future directions below.

### Caveats and future directions

As demonstrated, the demographic data to which researchers have access may limit the ways they are able to explore racial self‐categorization as an outcome. Although we reviewed several common approaches to racial self‐categorization data collection in the current manuscript, we recognize that there are other strategies to collecting such data via surveys. One approach which we did not address is to ask about the race of participants' parents and/or grandparents. Having more precise data about racial ancestry might pose additional possibilities for analyzing Multiracial adolescents' racial self‐categorizations, and perhaps certainty in the classification and identification of Multiracial samples. We identified one empirical study that leveraged racial ancestry data to explore racial self‐categorization among Mulitracial youth using multinomial logistic regression (Bratter, 2007). This suggests that some of the methodological recommendations we propose may also be useful for studying data that extends beyond our described scenarios.

Multiple caveats for applying our recommendations arise from sampling issues. One is that Multiracial people may not always self‐categorize with multiple racial groups on demographic forms (Lam‐Hine et al., 2024). For example, a Biracial Black–White person may choose to exclusively identify as Black on demographic forms. This will consequently limit researchers in being able to accurately identify Multiracial samples and represent them quantitatively. Asking about parent and/or grandparent race might alleviate this conundrum and offer greater model accuracy. Another crucial issue pertains to limited sample sizes of youth with particular Multiracial combinations. As we have noted, the limited sizes of certain subgroups often lead to severely constraining researchers' attempts to represent the full spectrum of Multiracial youth experiences; many more meaningful subgroups are possible than are represented in the literature. The only solution to this is to collect data with large enough subsamples of specific Multiracial combinations, and this is often a question of availability of data at hand (for secondary analyses) or resources for more inclusive sampling at the outset of a study.

Furthermore, in order to examine such questions research teams require sufficient statistical training. Scholars who choose to embark on this work should not only be prepared to specify and run the models but also be ready to confidently confront statistical challenges that arise along the way. These types of analyses might require researchers to push disciplinary boundaries—indeed, they necessitate multidisciplinary teams—in order to advance critical quantitative methodologies that are theoretically and analytically sound.

Additionally, there is not much precedent in terms of guidance for modeling racial self‐categorization as an outcome. The research questions offered in this article are meant to serve as starting points to spark thinking about these novel methodological applications; reviewing all possibilities and alternatives to studying Multiracial racial processes was outside the scope of this article. Thus, there are unexpected roadblocks that likely arise in examining other phenomena with Multiracial youth populations that should also be addressed in future conceptual work. As the design of studies with and about Multiracial youth continues to evolve, we expect that the “old” challenges presented herein will be replaced with new ones, as is often the case with the evolution of fields of inquiry.

## CONCLUSION

Rooted in QuantCrit and Quant(Multi)Crit perspectives, this article offers conceptual and practical guidance for scholars across disciplines interested in Multiracial youth and demonstrates how different analytic approaches allow researchers to consider racial self‐categorization as an outcome. These constructive insights support the field in advancing critical discourse that challenges “race‐neutral” methodologies (Castillo & Gillborn, 2022; Trawalter et al., 2020), improving our understandings and ultimately the development of Multiracial adolescents, and supporting the creation of more equitable and accurate survey‐based research. Such advancements are pressing and necessary given the rapidly growing Multiracial youth population. As this population continues to increase globally, researchers will find it increasingly hard to justify the use of monoracist statistical practices that collapse or erase Multiracial experiences. Instead, we encourage scholars to embrace novel applications of statistical approaches to studying complex populations in pursuit of more robust and accurate findings in regard to Multiracial lived experiences.

In addition to the issues raised in this article, we suspect that there will be ongoing, novel difficulties that arise in the field. However, scholars should not be deterred by these challenges. Studying Multiracial adolescents' racial self‐categorization may be challenging, but it is not impossible. In fact, we argue that the challenges that arise in doing this work underscore its importance. It demonstrates that conducting critical quantitative work aimed at representing complex lived experiences, while messy, is worth doing as it has the potential to challenge (mono)racism and more holistically honor the lived experiences of an underrepresented population in adolescent development research. Across disciplines, we need to build on existing contributions to date while better preparing the next generation of critical quantitative researchers to tackle new empirical challenges in pursuit of social justice (Frisby, 2024; Grogan‐Kaylor, 2025). Critical quantitative researchers should be both sufficiently versed in analytic approaches and have the vision to push statistical boundaries in ways that honor participant realities—in this case, those of Multiracial adolescents—to enrich our understanding of adolescent development.

## AUTHOR CONTRIBUTIONS

**Victoria Vezaldenos:** Supervision; conceptualization; methodology; writing – review and editing; writing – original draft. **Andrew Grogan‐Kaylor:** Conceptualization; writing – original draft; writing – review and editing; methodology. **Deborah Rivas‐Drake:** Writing – review and editing; conceptualization; writing – original draft.

## FUNDING INFORMATION

The authors have nothing to report.

## CONFLICT OF INTEREST STATEMENT

The authors declare no conflicts of interest.

## ETHICS STATEMENT

The authors have nothing to report.

## CONSENT

The authors have nothing to report.

## Data Availability

Data sharing not applicable to this article as no datasets were generated or analysed during the current study.

## Footnotes

^1^
Sometimes also referred to as self‐classification (see Roth, 2016).

^2^
In this manuscript, the terms ethnicity or ethnic identity are subsumed within race or racial identity unless otherwise noted. While we acknowledge that ethnicity and race are defined (and sometimes measured) distinctly, we recognize that people do not experience their ethnic and racial identities separately (Rivas‐Drake & Umaña‐Taylor, 2019; Schwartz et al., 2014). This is particularly relevant to ethnic groups like Latinxs and Arab, Middle Eastern, and North Africans in the U.S., as they are continually categorized and essentialized as members of a distinct group separate from other recognized racial categories (e.g., Black, White, Asian; D'Urso, 2026). By conceptualizing ethnicity and race as interlocking constructs, we align ourselves with dominant Multiracial discourse which considers ethnic group membership in defining who is Multiracial (e.g., Latinx–White, Middle Eastern–Black and similar combinations would be considered Multiracial; Atkin et al., 2022).

^3^
While outside the scope of the current manuscript, it is important to note that recent scholarship in adjacent fields has also explored how monoracial populations navigate self‐categorization (for instance, see Agadjanian, 2022; Agadjanian & Lacy, 2021; Egan, 2020; Roth, 2016) particularly amidst shifting definitions of racial and ethnic group boundaries (Brodkin, 1998; Lee & Ramakrishnan, 2020; Omi & Winant, 2008).

^4^
Indeed, critical quantitative approaches have been leveraged to examine self‐categorization among monoracial groups (see Phan & Lee, 2022).

^5^
Historically, AMENA has not been recognized as an ethnic group (D'Urso, 2026), and ethnicity and race have been prompted using two separate questions (U.S. Census Bureau, 2024a).

^6^
Importantly, monoracial groups might also select “Other” when they feel like their identity has not been accurately represented in the presented survey categories (see Brodkin, 1998; D'Urso, 2026).

^7^
Extensive scholarship has documented that the particular combination of Multiracial identity groups one belongs to has implications on their racialized experiences (Felkey, 2023; Reece, 2019; Song et al., 2022; Vezaldenos et al., 2025). With the “Biracial/Multiracial” category approach, however, one would be limited in their ability to explore such differences. In order to combat this issue, some researchers opt to program their surveys so that after one selects the “Biracial/Multiracial” category, they see an additional question that prompts them to specify which racial groups they identify with in more detail. This approach requires additional survey logic to be planned at the outset of data collection, thus demonstrating more intentionality in how to recognize Multiracial samples. Still, this approach may not be the most effective in capturing all Multiracial respondents, as not all people who hold multiple racial identities identify with the terms Biracial or Multiracial (Rockquemore, 1998; Root, 2003). For these respondents, they may prefer to select just one identity rather than selecting the “Biracial/Multiracial” category.

^8^
As a baseline, it is useful to consider a situation that may become increasingly less common in contemporary research but nevertheless prevalent in older or administrative datasets. This involves offering mutually exclusive racial category choices and asking youth to select only one. In this case, the investigative team might not have considered or anticipated the possibility of Multiracial participants at the outset of the study. In the data collection phase, participants were offered mutually exclusive racial category choices and asked to select just one that applied to them. As previously discussed, this approach certainly perpetuates monoracism, as it only recognizes singular, mutually exclusive racial labels as being valid responses. With no alternative means of reporting their racial self‐categorizations, Multiracial adolescents who complete the survey are made invisible. As the researchers would have no way of knowing which participants were actually Multiracial, during the analysis phases of the project, youth who should otherwise be considered Multiracial would unknowingly be included in the monoracial groups, likely introducing error. Accordingly, it is *not possible* to study the racial self‐categorization choices of Multiracial youth in this dataset because there is no way to identify who is actually Multiracial.

^9^
Note, we only discuss scenarios that have a data structure suitable for the particular analytic approach. So not every analytic approach will be relevant for both presented scenarios.

^10^
Some may choose to move ahead by estimating the predictors of this dichotomous dependent variable with linear regression, a practice that is termed a linear probability model (Agresti & Finlay, 1999). However, while providing intuitively simple results, linear probability models may result in some difficulties. First of all, the standard errors and hence the *p* values from a linear probability model will not be appropriate unless they are statistically adjusted. Second, the functional form of a linear model is often not considered appropriate, as the outcome of a linear probability model is allowed to vary from −∞ to +∞ while a dichotomous dependent variable is constrained to be either 0 or 1 (Grogan‐Kaylor, 2025). Lastly, a linear regression assumes a constant marginal association of the independent variable with the dependent variable (Grogan‐Kaylor, 2025).

^11^
Thus, models that incorporate multiple equations, and that allow for possible correlations of the errors across these equations, such as structural equation models (StataCorp, 2025), would be an important improvement upon these methods. Structural equation models are discussed later in this manuscript.

^12^
If those outcomes are considered to be ordered, an ordinal logistic regression model (StataCorp, 2025) may be appropriate. However, racial or ethnic identification has no underlying order; therefore, we do not discuss ordinal logistic regression in any greater detail here.

^13^

*Note*. The equations are estimated separately and simultaneously. β coefficients in each equation have different values. For the sake of parsimony and simplicity, and to avoid overcomplicating the notation, they are all presented as β.

^14^
Note, scenario 2 only contained “choose one” data with options for “Other” and “Multiracial.” This means that in a multinomial logistic regression where a categorical outcome variable with three or more options is necessitated, researchers would need to compare Multiracial and Other respondents to at least one of the monoracial categories presented. These comparisons are not recommended; thus, the “choose one” data from scenario 2 would not be suitable for examination with this methodological approach.

^15^
Another type of qualitative change to self‐categorization that may be of substantive interest is respondents who indicated three or more check boxes for their racial identity but then reduced those selections at a later time point (i.e., consolidation; see Hitlin et al., 2006). This may also be explored cross‐sectionally in the case of scenario 1 such that a participant may indicate multiple response options like “Asian,” “Black,” and “White,” but then when asked to choose one they could indicate “Other” and write in only “Asian” and “Black.” These particular cases can be modeled as an outcome of interest, but the number of cases that meet these criteria may be small. Thus, these circumstances may only be feasible to explore with larger, population‐level datasets.

^16^

*Note*. The equations are estimated separately and simultaneously. *β* coefficients in each equation have different values. For the sake of parsimony and simplicity, and to avoid overcomplicating the notation, they are all presented as β.

## APPENDIX A Author Positionality Statements

The first author, Victoria Vezaldenos, identifies as Biracial Mexican and White. Her own lived experiences navigating monoracism have motivated her continued interests in studying Multiracial experiences holistically. Victoria is completing her graduate training at the University of Michigan, an elite public university, within a program that emphasizes rigorous methodological training. Early in her studies, she developed an interest in leveraging quantitative methods to study issues related to racial identity development. Through this coursework, challenges inherent to the study of Multiracial groups became apparent. As a result, Victoria has worked to align her methodological training with her scholarly interests that center Multiracial youth while also working toward antiracism. Thus, these intersections of her academic training and personal experiences continue to shape her approaches to quantitative methods as she strives to offer novel perspectives that may result in more accurate representations of Multiracial communities in quantitative scholarship from across disciplines.

The second author, Dr. Andrew Grogan‐Kaylor, identifies as white. His experiences growing up in a family with limited socioeconomic resources inculcated an early interest in using quantitative methods to examine disparities and inequalities of all kinds. Dr. Grogan‐Kaylor is the Sandra K. Danziger Collegiate Professor of Social Work at the University of Michigan. His current work focuses on the use of multilevel models to explore parenting and child development with international data, and his recent reading on critical quantitative approaches has suggested such approaches might provide theoretical guidance for his future work.

The third author, Dr. Deborah Rivas‐Drake, is a cisgender Latina second‐generation child of working‐class immigrants who uses asset‐based approaches to frame her scholarship. She aims to work in solidarity with others—including her children with a White, non‐Latinx partner—who experience various intersectional oppressions that do and do not mirror hers to continuously advance our collective emancipatory agency. As a scholar trained in quantitative, qualitative, and mixed‐method paradigms, she seeks to employ methodological and analytic approaches— including critical quantitative and QuantCrit lenses—that humanize and reflect the complexity of the lived experiences of youth, families, and practitioners in her studies. She views this endeavor as a career‐long undertaking, and she feels fortunate to learn alongside her colleagues, trainees, and students in doing so.
